# Exploring colorectal cancer patients’ diagnostic pathways and general practitioners’ assessment of the diagnostic processes: a Danish survey study

**DOI:** 10.1080/02813432.2024.2432376

**Published:** 2024-11-25

**Authors:** Dorte E. Jarbøl, Sanne Rasmussen, Kirubakaran Balasubramaniam, Jesper Lykkegaard, Linda Juel Ahrenfeldt, Gitte B. Lauridsen, Peter Haastrup

**Affiliations:** a Department of Public Health, Research Unit of General Practice, University of Southern Denmark, Odense M, Denmark; b Audit Project Odense, Research Unit of General Practice, University of Southern Denmark, Odense M, Denmark

**Keywords:** Colorectal cancer, General practice, Diagnosis, Evaluation, Symptoms

## Abstract

**Introduction:**

Colorectal cancer (CRC) is among the most common cancers and the prognosis of CRC is highly dependent on stage at diagnosis. Although many cases are diagnosed swiftly, there is still room for improvement.

**Aim:**

We aimed to explore CRC diagnostic pathways, encompassing (1) place of initial contact; (2) associations with symptom presentations, sex, and age with events in the diagnostic process and initial referrals and (3) the general practitioner’s (GP’s) evaluation of the diagnostic processes.

**Methods:**

All GPs in North-, Central-, and Southern Denmark were invited to fill in questionnaires for their listed patients diagnosed with cancer during the past two years.

**Results:**

Among 1,032 recorded CRC patients, 65% had their initial contact in general practice, 5% within the out-of hours service, 10% in the hospital, and 20% were diagnosed based on screening. A total of 27% of CRC patients over 40 who initially presented in general practice were treated or referred on suspicion of another disease first, and 9% were reported to have had hesitated in seeking medical attention. Some 37% presented solely non-specific symptoms, increasing the odds of the GP advising watchful waiting (OR 2.48; 95% CI 1.06–5.81), treating or referring on the suspicion of another illness first (OR 2.57; 95% CI 1.76–3.75), wait due to normal findings (OR 2.11; 95% CI 1.16–3.85), or referring to diagnostic imaging (OR 3.07; 95% CI 1.63–5.79). The GPs assessed nearly one fifth of the diagnostic processes as poor.

**Conclusion:**

Most CRC patients are diagnosed with initial presentation in general practice. Having non-specific symptoms is common and challenges timely diagnosis.

## Introduction

Colorectal cancer (CRC) stands as a significant global health concern due to its high incidence and mortality rates [1]. The prognosis of CRC is highly dependent on stage at diagnosis, and despite efforts to expedite the diagnostic process, most CRC cases are unfortunately still diagnosed in advanced stages, where curative treatment is hampered [2]. In many countries, CRC screening programmes have been established, but most cases of CRC are still to be diagnosed based on symptom presentation [3]. Symptoms such as rectal bleeding, changes in bowel habits, and abdominal discomfort are well known alarm symptoms of CRC; however, the positive predictive values are low – i.e. the symptoms are in most cases, caused by benign conditions [4]. Therefore, the diagnosis can be challenging for the general practitioner (GP), who often is the patients’ initial contact in the diagnostic process and hence responsible for the initial assessments and referral for further investigations [5]. Moreover, it is known that the risk of an increased diagnostic primary care interval is substantially higher for patients where the GP does not initially suspect cancer [6]. Although a recent study has shown that the number of contacts to general practice increase prior to diagnosis [7], barriers for healthcare seeking are common among individuals experiencing bowel symptoms [8], challenging timely diagnosis of CRC. Demographic factors such as age and sex have been observed to influence both the interpretation of symptoms by patients and healthcare professionals and the subsequent decision-making processes, potentially contributing to delays in diagnostic procedures [9]. Previous studies have demonstrated that the GP most often is involved in the diagnostic process of cancer [5,6,10] but healthcare seeking with symptoms may change over time [11]. How the role of Danish GPs in diagnosing CRC may have changed during the past decade, where the national CRC screening program for citizens aged 50–74 years was enrolled, has not previously been studied.

With the GP being a cornerstone in the diagnostic process for many CRC patients [12], the GP’s evaluation of the diagnostic processes is important to gain insight into. The GP has a unique position to evaluate the overall diagnostic process, as the GP is the only person in the healthcare system with access to information about the entire diagnostic process from initial symptom presentation to diagnosis [13].

To inform targeted interventions and policies aimed at improving the efficiency and effectiveness of CRC diagnostic pathways, a broader understanding of the diagnostic processes in general practice is warranted.

Thus, the aim was to explore CRC patients’ diagnostic pathways, in this study encompassing 1) the place of initial contact with the healthcare system; 2) the influence of symptom presentations, sex and age on the occurrence of events in the diagnostic process and which other part of the healthcare system the GP first refers to and 3) exploring the GP’s evaluation of the diagnostic processes.

## Methods

### Data sources and study population

This study was based on data from a survey carried out in 2021 in the Regions of Southern, Northern and Central Denmark, which comprise approximately 3,134,000 inhabitants, corresponding to 54% of the entire Danish population. All 852 general practices within the three regions were invited to participate in the survey, which was based on the Audit Project Odense method [14]. The aim of the survey was to explore the diagnostic process, including symptom presentation and duration, diagnostic procedures in and referrals from general practice, and the GP’s assessment of the overall diagnostic trajectory for incident cancer patients. The survey comprised a retrospective review of medical records and the GPs’ recollection of the diagnostic processes for patients listed with the GP. Among the questions in the survey, the GP assessed the occurrence of diagnostic events in the diagnostic process. The invitation and survey are described in detail elsewhere [15].

Patients relevant for the study were identified through the region’s administrative databases and included patients diagnosed with incident cancer according to International Classification of Diseases version 10. Cancer codes C00–C99 were considered (excluding nonmelanoma skin cancer C44). The study included all incident cancers, diagnosed between 1 March 2019 and 28 February 2021, and listed with a participating general practice. Patients were excluded if registered with a cancer diagnosis five years prior the diagnosis.

The regions’ administrative databases are the databases for diagnoses transferred to the National Patient Register, which is considered a valid source of information on diagnoses [16]. A list including cancer patients for the particular general practice was sent to each participating GP for further verification. This study is one of a series of papers and is based on GP-reported data for all CRC patients in the study population. Detailed information about the data collection procedure can be found elsewhere [15].

### Setting

In Denmark, almost all citizens are listed with a GP, who performs the initial assessment of patients presenting symptoms, referring for specialised diagnostics, coordinating care, managing chronic diseases, and collaborating with both primary and secondary care specialists. The GP thus serves as a gatekeeper and has the broadest overview of the patient within the healthcare system. GP services are tax-funded for all citizens [13].

### The questionnaire

The questionnaire’s conceptual framework comprised an investigation of the diagnostic process for the incident cancer patients in each practice. The diagnostic process comprised time and events from initial contact to the first referral and the GPs’ retrospective evaluation of each diagnostic process [15].

Questions capturing the following aspects of the CRC diagnostic process were included in this study: Place of the patient’s initial contact with the healthcare system with signs or symptoms that could be caused by CRC, symptom presentation, events in the diagnostic process, the first referral for further investigation and the GP’s overall assessment of the diagnostic process.

For patients with initial contact in general practice, the responding GPs indicated whether the patient presented with CRC-specific symptoms, non-specific symptoms, or no symptoms (symptom list given in Supplementary Table 1). Absence of symptoms could indicate that the patient had contacted the GP with other issues or that the suspicion of cancer was caused by incidental findings in patients with no related symptoms.

Place of initial contact in the diagnostic process was categorized as follows: (1) general practice, (2) out-of-hours service, (3) hospital (including emergency call, outpatient clinic) and (4) patients diagnosed based on the national screening program for CRC, unknown place of contact, or missing.

Events in the diagnostic process in general practice comprised events related to either the patient or the GP, i.e. the patient (1) hesitated to contact the GP, (2) did not want investigation and (3) did not comply with the follow-up agreement, or the GP (1) advised watchful waiting without a timeframe, (2) initially treated or referred on suspicion of another disease, (3) waited with further investigations because of a normal examination and (4) initially referred for investigation of another cancer type than CRC.

The first referral from general practice was reported as (1) a cancer patient pathway (CPP), (2) the Cancer Patient Pathway for Non-specific Symptoms and Signs of Cancer (NSSC-CPP), (3) diagnostic imaging, (4) medical specialist or other outpatient clinic and (5) acute hospital admission.

The GPs’ evaluation of the diagnostic processes encompassed the assessment of the overall diagnostic process and the four subcategories (the patient’s, the GP’s, and the secondary sector’s role in the process, and the transition between primary and secondary care) on a four-point Likert scale: (1) ‘very good’; (2) ‘predominantly good’; (3) ‘predominantly poor’; and (4) ‘very poor’. For analyses, responses were dichotomised into (1) ‘overall good’ (very good/predominantly good) and (2) ‘overall poor’ (very poor/predominantly poor).

All variables used in the study, with the exact wording, response scales, variable coding, and data sources are available in Supplementary Table 2.

### Analyses

Differences in place of initial contact between sexes, age groups and regions were tested using the Chi-square test.

**Figure 1.**
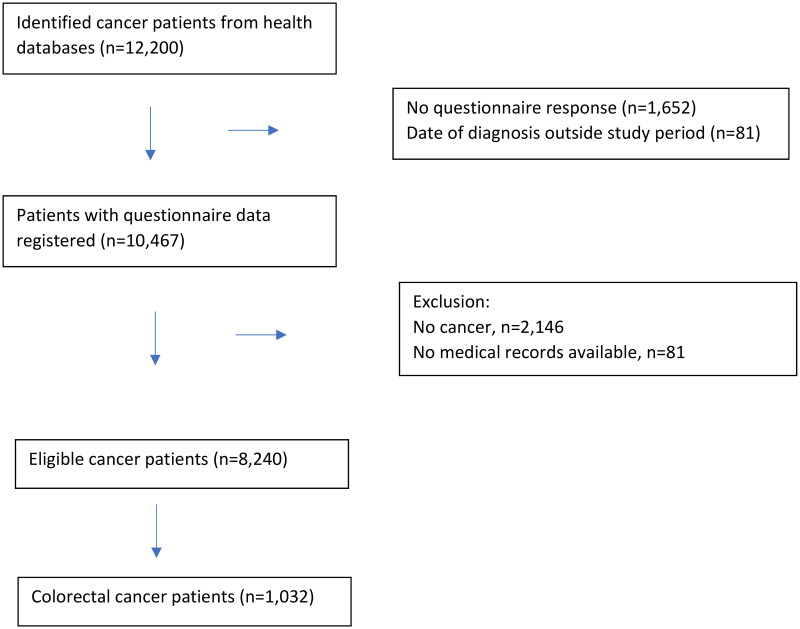
Flow chart.

For the outcome variables ‘events in the diagnostic process’ and ‘initial referral’, we only included cancer patients over 40 years, as this is the recommended age cutoff above which the clinician should be especially aware of colorectal cancer, and patients where general practice was the patients’ initial contact with the healthcare system (Figure 1). Explanatory variables comprised (1) the patient’s age and sex, and (2) first symptom presentation (‘only specific cancer symptoms’, ‘only non-specific symptoms’, ‘both specific cancer and non-specific symptoms’ and ‘no symptoms’). The explanatory variables were chosen because each of these were considered to be associated with both events in the diagnostic process and first referral. Descriptive data are given as numbers and percentages.

Associations between the explanatory variables and the events in the diagnostic process and different categories of first referral were investigated using regression analyses with robust standard errors clustered by each general practice, adjusted for age, sex and symptom presentation, and reported as odds ratios (ORs) with 95% confidence intervals (CIs).

The GPs’ assessment of both the overall diagnostic process as well as the role of the patient, the GP and the secondary care sector for each incident cancer patient with initial contact in general practice were described for the CRC-patients and for all cancer patients, but not CRC. Chi-square tests were performed for difference between the two groups.

## Results

A total of 187 general practices (21%) answered the questionnaire. From corresponding administrative databases based on hospital records, a total of 12,200 patients were registered with a new cancer diagnosis in the study period, and the GPs had filled in complete questionnaire data regarding 10,467 patients. Excluding patients not meeting inclusion criteria resulted in a sample of 8,240 cancer patients, of which 1,032 (12.5%) were diagnosed with CRC (Figure 1).

Characteristics of the study sample and initial contact with signs or symptoms that could be caused by CRC are presented in Table 1. A total of 675 (65%) of the patients had their initial contact in general practice; 54 (5%) had their initial contact within the out-of-hours service; 100 (10%) were diagnosed based on initial contact within the hospital and 203 (20%) were diagnosed based on screening (*n* = 193)/other pathways (*n* = 10).

**Table 1.** Study population.

	All CRC patients *N* (%)	Patients with initial contact in general practice *N* (%)	Patients with initial contact within the out-of-hours service *N* (%)	Patients with initial contact within the hospital, including emergency call/112, outpatient clinic *N* (%)	Patients diagnosed based on screening (*n* = 193)/others (*n* = 10) *N* (%)	*p*-Value**
Total	1032	675 (65.4)	54 (5.2)	100 (9.7)	203 (19.7)	
Women	494 (100)	336 (68.0)	25 (5.1)	44 (8.9)	89 (18.0)	0.398
Men	538 (100)	339 (63.0)	29 (5.4)	56 (10.4)	114 (21.2)	
Age groups (yrs)						
0–40	32 (100)	22 (68.8)	6 (18.8)	*	*	<0.001
41–60	168 (100)	96 (57.1)	5 (3.0)	14 (8.1)	57 (33.1)	
61–80	590 (100)	361 (61.2)	22 (3.7)	64 (10.8)	143 (24.2)	
81+	242 (100)	196 (81.0)	21 (8.7)	22 (9.1)	3 (1.2%)	
Region						
The Region of Southern Denmark	387 (100)	244 (63.0)	22 (5.7)	38 (9.8)	83 (21.4)	0.497
The Central Denmark Region	522 (100)	349 (66.9)	24 (4.6)	55 (10.5)	94 (18.0)	
The North Denmark Region	123 (100)	82 (66.7)	8 (6.5)	7 (5.7)	26 (21.1)	

Characteristics of colorectal cancer (CRC) patients stratified on place of initial contact with symptoms attributable to later diagnosed CRC.

*An asterisk means that within that category, the values below five have been added to the nearest cell.

**χ^2^ test for difference between the groups.

Table 2 presents the characteristics of incident CRC patients over 40 years with initial contact in general practice, and the results of adjusted analyses of associations between patient characteristics, symptom presentation and events in the diagnostic process (crude ORs in Supplementary Table 3).

**Table 2.** Odd ratios for events in the diagnostic process according to age, sex and symptom presentation in 653 incident CRC patients above 40 years with initial contact in Danish general practice.

	All *N* (%)	The patient hesitated with healthcare-seeking	The patient did not want an investi-gation	The patient did not comply with follow-up agreement	The GP advised watchful waiting with no time indication	The GP treated or referred on the suspicion of another disease first	The GP waited with further investigations because of normal examina-tions	The GP referred to investi-gation on suspicion of another cancer type first
Total*	*N* = 653 (100)	*N* = 91 (13.9%)	*N* = 60 (9.2%)	*N* = 23(3.5%)	*N* = 26 (4.0%)	*N* = 177(27.1%)	*N* = 41 (6.3%)	*N* = 25 (3.8%)
		Adj. OR^a^ (95% CI)	Adj. OR^a^ (95% CI)	Adj. OR^a^ (95% CI)	Adj. OR ^a^ (95% CI)	Adj. OR^a^ (95% CI)	Adj. OR^a^ (95% CI)	Adj. OR^a^ (95% CI)
Sex								
Women	323 (49.5)	Ref	Ref	Ref	Ref	Ref	Ref	Ref
Men	330 (50.5)	0.94 (0.59, 1.48)	0.93 (0.54, 1.60)	**2.86 (1.03, 7.96)**	1.29 (0.55, 3.02)	0.73 (0.50, 1.06)	0.80 (0.41, 1.58)	0.83 (0.41, 1.71)
Age groups, years								
41–60	96 (14.7)	Ref	Ref	Ref	Ref	Ref	Ref	Ref
61–80	361 (55.3)	0.73 (0.38, 1.41)	1.19 (0.50, 2.79)	0.49 (0.16, 1.47)	0.98 (0.27, 3.57)	0.88 (0.48, 1.61)	1.32 (0.47, 3.66)	0.97 (0.30, 3.06)
>80	196 (30.0)	0.86 (0.47, 1.59)	**2.50 (1.06, 5.94)**	0.73 (0.23, 2.33)	1.97 (0.55, 7.04)	0.91 (0.48, 1.75)	1.15 (0.40, 3.30)	0.85 (0.23, 3.10)
Symptom presentation								
None	51 (7.8)	0.48 (0.18, 1.30)	0.35 (0.08, 1.60)	N/A	N/A	**0.23 (0.07, 0.78)**	0.39 (0.05, 3.10)	N/A
Only specific	312 (47.8)	Ref	Ref	Ref	Ref	Ref	Ref	Ref
Only non-specific	240 (36.8)	**0.49 (0.29, 0.83)**	0.73 (0.41, 1.30)	1.57 (0.59, 4.14)	**2.48 (1.06, 5.81)**	**2.57 (1.76, 3.75)**	**2.11 (1.16, 3.85)**	**5.91 (2.08, 16.80)**
Both	50 (7.7)	0.38(0.13, 1.09)	0.74 (0.24, 2.30)	1.91 (0.48, 7.53)	2.46 (0.60, 10.12)	1.36 (0.69, 2.67)	0.84 (0.20, 3.57)	**6.73 (1.71, 26.39)**

^a^ Adjusted for age, sex and symptom presentation.

* The number of the different events do not sum up to 100% as it was also possible to indicate none of the abovementioned events.

Bold indicates significance at 5% level

Among the patients with initial contact in general practice, 177 (27%) were treated or referred on suspicion of another disease first. The GPs reported that 91 (14%) of the patients had hesitated to seek medical attention, and in 41 (6%) of the processes, the GPs waited based on normal examination findings. Men had higher odds for not complying with the follow-up agreement compared to women (*OR*_adj_ 2.86; 95% CI 1.03–7.96) (Table 2). Moreover, more patients aged 80 years and older were more likely to decline an investigation compared to patients in the youngest age group (*OR_adj_* 2.50; 95% CI 1.06–5.94), (Table 2).

Compared to patients with specific symptoms only, presenting with a combination of specific and non-specific symptoms increased the odds of being referred to investigation on suspicion of another cancer first (*OR_adj_* 6.73; 95% CI 1.65–26.39). Presenting with only non-specific symptoms increased the odds of the GP to advise watchful waiting without time indication (*OR_adj_* 2.48; 95% CI 1.06–5.81), treat or refer on the suspicion of another illness first (*OR_adj_* 2.57; 95% CI 1.76–3.75), and wait due to normal findings (*OR_adj_* 2.11; 95% CI 1.16–3.85), but decreased the odds of the patients having hesitated to seek health care (*OR_adj_* 0.49; 95% CI 0.29–0.83), (Table 2).

Table 3 shows results related to the first referral in the diagnostic process among CRC patients over 40 with first presentation in general practice. A total of 49% were first referred to a specific CPP, and 15% were first acutely hospitalised. A total of 10% were first referred to diagnostic imaging, and 17% were first referred to a specialist or other hospital departments. Men were less likely to have been referred to specialist or other hospital departments first, compared to women (*OR_adj_* 0.68; 95% CI 0.46–1.00).

**Table 3.** Results of cluster regression analyses estimating the associations between age, sex and symptom presentation and the first referral in the diagnostic process, shown as adjusted odds Ratio^a^ (adj. OR) with 95% confidence intervals (CIs), in 653 incident CRC patients in Danish general practice above 40 years (2019–2021).

	Referred to a specific cancer patient pathway	Referred to a non-specific cancer patient pathway	Referred to diagnostic imaging	Referred to specialist or other hospital department	Acute hospitalisation
Total* *N* = 653	*N* = 320 (49.0%)	*N* = 35 (5.4%)	*N* = 68 (10.4%)	*N* = 108 (16.5%)	*N* = 95 (14.5%)
	Adj. OR^a^ (95% CI)	Adj. OR^a^ (95% CI)	Adj. OR^a^ (95% CI)	Adj. OR^a^ (95% CI)	Adj. OR^a^ (95% CI)
Sex					
Women	Ref	Ref	Ref	Ref	Ref
Men	1.28 (0.93, 1.75)	0.79 (0.41, 1.54)	1.33 (0.79, 2.22)	**0.68 (0.46, 1.00)**	0.96 (0.60, 1.54)
Age groups, years					
41–60	Ref	Ref	Ref	Ref	Ref
61–80	1.31 (0.76, 2.26)	2.17 (0.61, 7.77)	1.21 (0.54, 2.68)	0.76 (0.40, 1.45)	0.64 (0.34, 1.19)
>80	1.02 (0.58, 1.80)	1.55 (0.39, 6.24)	1.37 (0.55, 3.40)	0.52 (0.25, 1.10)	1.41 (0.72, 2.76)
Symptom presentation					
None	**0.38 (0.22, 0.65)**	**3.51 (1.15, 10.64)**	0.94 (0.26, 3.38)	1.08 (0.51, 2.27)	1.94 (0.91, 4.15)
Only specific	Ref	Ref	Ref	Ref	Ref
Only non- Specific	**0.27 (0.19, 0.38)**	2.42 (0.95, 6.13)	**3.07 (1.63, 5.79)**	1.13 (0.74, 1.72)	**2.65 (1.54, 4.57)**
Both	0.74 (0.42, 1.31)	**3.86 (1.24, 12.02)**	0.93 (0.28, 3.09)	0.85 (0.44, 1.63)	1.63 (0.69, 3.85)

^a^ Adjusted for age, sex and symptom presentation.

*The number of the different processes do not sum up to 100% as it was also possible to indicate none of the abovementioned processes.

Bold indicates significance at 5% level

Patients presenting with non-specific symptoms were more likely to be referred to diagnostic imaging (*OR_adj_* 3.07; 95% CI 1.63–5.79), and for acute hospitalisation (*OR_adj_* 2.65; 95% CI 1.54–4.57), compared to patients presenting with specific CRC symptoms. Patients presenting with both specific and non-specific symptoms were more likely to be referred to a NSSC-CPP (*OR_adj_* 3.86; 95% CI 1.24–12.02). Presenting without symptoms or non-specific symptoms decreased the odds for referral in a CPP (Table 3). (Crude ORs in Supplementary Table 4.)

Results indicated that the GPs’ assessment of their patients’ cancer diagnostic processes in general was assessed slightly more negatively than the GP’s role, the transition between sectors and the secondary sector’s role (Table 4). Overall, most diagnostic processes were assessed as good or predominantly good among all cancer patients and the CRC patients, though the assessment among CRC patients was slightly more negative when comparing to all cancer patients, which is statistically significant for the overall assessment and the assessment of the patient’s role (Table 4).

**Table 4.** The general practitioners’ assessment of their patients’ cancer diagnostic processes^a^.

	All cancer patients, but not CRC, with initial contact in general practice, *n* (%)	CRC patients with initial contact in general practice, *n* (%)	*p*-Value*
Overall assessment	5,233 (100)	675 (100)	0.043
Very good/predominantly good	4,586 (87.6)	574 (85.0)	
Very poor/predominantly poor	604 (11.5)	96 (14.2)	
Missings	43 (0.8)	5 (0.7)	
Assessment of the patient’s role			<0.001
Very good/predominantly good	4,565 (87.2)	548 (81.2)	
Very poor/predominantly poor	611 (11.7)	121 (17.9)	
Missing	57 (1.1)	6 (0.9)	
Assessment of the GP’s role			0.186
Very good/predominantly good	4,872 (93.1)	620 (91.9)	
Very poor/predominantly poor	319 (6.1)	50 (7.4)	
Missing	42 (0.8)	5 (0.7)	
Assessment of the sector transition			0.393
Very good/predominantly good	4,878 (93.2)	635 (94.1)	
Very poor/predominantly poor	306 (5.8)	34 (5.0)	
Missing	49 (0.9)	6 (0.9)	
Assessment of the secondary sector’s role			0.680
Very good/predominantly good	4,801 (91.7)	623 (92.3)	
Very poor/predominantly poor	347 (6.6)	42 (6.2)	
Missing	85 (1.6)	10 (1.5)	

*χ^2^* test for difference between the two groups.

^a^Questionnaire data of the GPs’ assessment of both the overall diagnostic process as well as the role of the patient, the GP and the secondary care sector for each incident cancer patient within the practice between 1 March 2019 and 28 February 2021.

## Discussion

### Main findings

Our findings underline that most CRC patients are diagnosed outside the screening programme and with initial contact outside the hospital sector, i.e. general practice. The results showed that patients experiencing non-specific symptoms only were less likely to have hesitated with seeking health care. This may indicate that the patients reacted relevantly on feeling unwell. However, non-specific symptoms seem to provide a challenge for the GPs since the odds of possible delaying factors, such as referral to investigation on suspicion of another cancer type first, were all higher when the patients only experienced non-specific symptoms. Most CRC diagnostic processes were assessed as good by the GPs, but in nearly one fifth of the cases, the patient’s role in the process was judged as poor by the GP.

### Strengths and limitations

Incident CRC patients were included in the study from the regions’ administrative databases, which are considered to have a high completeness regarding cancer patients [17]. Furthermore, the cancer diagnoses were verified by the GPs. Although the study population is large, participation in the study was voluntary, and only 21% of eligible GPs participated. Therefore, selection bias cannot be ruled out. Moreover, the decision to participate in the study could be due to a special interest in cancer patients and a desire to improve the management of these patients in one’s practice [14]. This could potentially cause an underestimation of the number of patients with a poor overall diagnostic process if the missing questionnaire data were comprised of individuals with difficult or uneven diagnostic processes. Furthermore, the GPs’ assessments of the overall diagnostic process were subjective and retrospective. Although the GPs were encouraged to provide their honest assessments, it cannot be ruled out that some may have retrospectively viewed diagnostic processes as suboptimal when the suspicion of CRC was not raised during the first consultation, even though the fastest diagnostic processes are not always the most favourable [18].

The data on each patient were extracted from questionnaires filled in by a GP from the practice where the patient was listed, but not necessarily a GP whom the patient had consulted during the process. The questionnaire was pilot tested to ensure high content validity. Data were extracted from the available medical records; hence, only information noted in the patient’s file or recollected by the GP was included leading to a possible underreporting of events during the diagnostic process. Further, recall bias may exist, and the fact that the information was GP assessed might be a limitation for some of it. Taking patients who hesitated to contact their GP as an example, we cannot know to what extent this information was reliably available to the GP. Consequently, hesitation to contact the GP might be underestimated.

We found few CRC patients with both specific and non-specific symptoms (7.7%), which means that underreporting of non-specific symptoms is possible. This could be attributed to an increased emphasis on recording cancer-specific symptoms relevant to a particular cancer diagnosis or a missing documentation of non-specific symptoms when a patient also presents with a specific symptom. Underreporting of combination of both specific and non-specific symptoms may introduce underestimation of the odds of being referred to investigation on suspicion of another cancer first. Overall, information bias cannot be ruled out.

The GPs attended the study as a part of their voluntary continuous education with the aim of improving the quality of their own practices. Therefore, it seems fair to assume that they provided information they found to be accurate and valid.

We applied a five-year look-back period to classify cancers as incident, accepting initial mislabelling as incident of any relapsing CRC after a five-year period, without any hospital encounter for the disease. However, this mislabelling was likely corrected by the GPs.

Finally, we did not include information about neither comorbidity nor socioeconomic status, which might have confounded the associations between symptom presentation and the diagnostic processes.

### Discussion of results/comparison with existing literature

Our findings underline that most CRC patients are diagnosed outside the screening programme and with initial contact in general practice. Danckert et al. did also explore cancer patient pathways but based on register-data [19]. Their study found that the most common routes to diagnosis were cancer patient pathway from primary care, but only 46% of the patients were referred from general practice, which is likely due to a lack of data from general practice.

Previous studies have shown that bowel symptoms can be difficult for patients to talk to their GP about [8], and that patients with bowel symptoms may be reluctant to seek medical attention because they fear of unpleasant or embarrassing investigations [20,21] The fact that CRC patients with unspecific symptoms were less likely to hesitate in seeking health care than CRC patients with specific symptoms could indicate that specific CRC symptoms is still associated with more barriers towards help seeking [22]. From population studies, we know that bowel symptoms are common [23]. This can also create challenges for patients who exhibit only specific CRC symptoms, as the normality of some of these symptoms may make it difficult for them to determine when to contact their GP [20].

The fact that many patients present with vague and unspecific symptoms is also supported in previous findings [5]. Despite specific symptoms being common among CRC patients, more than a third of the CRC patients had only reported non-specific symptoms, challenging the GP in timely diagnosis [20]. Among the patients with only non-specific symptoms the odds of advising watchful waiting or the GP waiting with further investigation because of normal examinations were increased, i.e. the CRC can be hard to suspect when no specific symptoms are present [24]. This is also supported by our finding that patients with only non-specific symptoms had higher odds of being referred to diagnostic imaging or being admitted acutely and aligns with a study from the Netherlands that explored the diagnostic process related to the interpretation of symptoms and the GPs’ initial referral. The study showed that almost half of the patients initially presented with ‘non-alarm gastrointestinal symptoms’ or ‘other’ symptoms, which were associated with a long duration of the diagnostic process. Several themes related to postponed referral were identified, but the dominant theme was ‘having an alternative working diagnosis’, with the leading subtheme being ‘the presence of an explanatory concomitant condition’. The second main theme was ‘suboptimal diagnostic strategies’, with subthemes ‘omitting to reconsider an initial diagnosis’ and ‘lacking follow-up’. [25].

We cannot know why the GPs more often assessed the overall diagnostic process as very poor or predominantly poor among CRC patients compared to other cancer patients’ diagnostic processes, but it might be that the high proportion of CRC patients presenting only non-specific symptoms, coupled with the increased occurrence of events that could potentially delay timely diagnosis, may represent a critical concern [26].

## Implications

Our findings are important and should be communicated to GPs and healthcare planners because general practice plays an important role in diagnosing CRC, as general practice is the most common place of initial contact in the diagnostic process. Despite implementation of the CRC screening programme, it is important to remember that most CRC patients are diagnosed due to symptom presentation.

Further, it is important knowledge for clinicians that a third of the CRC patients exhibit only non-specific symptoms which complicates the timely diagnosis of CRC. Patients who solely exhibit non-specific symptoms are more often referred to the NSSC-CCP, which often consists of investigation with CT of thorax and abdomen without bowel preparation. However, this diagnostic modality is insufficient in diagnosing as well as ruling out CRC [27]. Therefore, it is important for GPs and physicians evaluating patients in the non-specific cancer patient pathway to consider whether a supplemental FIT test or a colonoscopy is necessary to rule out CRC in patients with non-specific symptoms despite normal CT scan. Whether this is relevant should be carefully considered in each patient to avoid overdiagnosis and unnecessary colonoscopies that are unpleasant for the patient and even may cause iatrogenic harm [28,29]. In the Danish guidelines for the Non-specific Symptoms and Signs of Cancer-Cancer Patient Pathway, FIT-testing for patients with no CRC symptoms has been implemented as a possibility to assist in deciding which patients with only non-specific symptoms of cancer should be offered a colonoscopy.

The healthcare providers may anticipate that patients with CRC will receive their diagnosis either through general practice or national screening programmes. However, it is noteworthy for future healthcare planning that a subset of CRC patients is diagnosed based on their initial contact with hospitals, as also shown for other cancers [30]. Additionally, some patients who initially seek care from general practice end up being acutely hospitalised [31]. This underscores the importance of continued efforts to enhance timely diagnosis of CRC within primary care settings.

## Conclusion

In this study, we found that general practice most often is the initial point of contact in the diagnostic process of CRC. Having only non-specific symptoms of CRC is common and potentially challenges timely diagnosis and is associated with being referred to diagnostic imaging, acute admission, or a NSSC-CCP where colonoscopy is not necessarily part of the investigation programme.

Most CRC diagnostic processes are assessed as good by the GPs but in nearly one fifth of the cases the patient’s role in the process is judged as poor by the GP, indicating that it can be challenging for patients to recognise CRC symptoms as relevant for seeking health care and that they may find it embarrassing to discuss bowel symptoms with their GP.

## Supplementary Material

supplementary Tables.docx

## Data Availability

The datasets generated and analysed in the current study are not publicly available and cannot be shared due to the data protection regulations promulgated by the Danish Data Protection Agency. Access to data is strictly limited to the researchers who have obtained permission for data processing. This permission was granted to the Research Unit of General Practice, Department of Public Health, University of Southern Denmark. Further inquiries can be made to PI Jesper Lykkegaard (email: jlykkegaard@health.sdu.dk).
