# Supplementary material for: Exploring colorectal cancer patients’ diagnostic pathways and general practitioners’ assessment of the diagnostic processes: a Danish survey study
Source: Scand J Prim Health Care. 2024 Nov 25;43(2):303–12. doi: 10.1080/02813432.2024.2432376 (PMC12090287; doi:10.1080/02813432.2024.2432376)
Supplement: supplementary Tables.docx [file IPRI_A_2432376_SM8160.docx]

**Supplementary Table 1 – Symptom list**

**Definition of symptom categories**

***General alarming symptoms or signs: Symptoms or signs that may be due to serious illness but which in themselves do not prompt an organ-specific cancer patient pathway, e.g.(1):***

Weight loss

Loss of appetite

Fever without known origin

Night sweats

Increased tendency of infection

Swollen lymph node

Anaemia

***Non-specific symptoms: Frequent, general symptoms, that on their own do not cause suspicion of serious illness, e.g.(1):***

Fatigue

Nausea

Bloating/abdominal distention

Concentration difficulty

Memory difficulty

Malaise

Discomfort

Non-specific pain

Lack of energy

Vertigo

Headache

Back pain

Swollen legs

***Specific colorectal cancer symptoms and signs: Symptoms and signs mentioned in organ-specific cancer patient pathway that prompt a cancer patient pathway (2).***

Age >40 years with at least one of the following symptoms:

- Visible rectal bleeding (fresh/not fresh)
- Changed bowel habits for >1 month
- Iron deficiency anaemia

Significant general symptoms, e.g., weight loss, abdominal pain

1. The National Board of Health. Diagnostisk pakkeforløb [In Danish]. www.sst.dk: Sundhedsstyrelsen; 2022.

2. The National Board of Health. Oversigt over kræftpakkeforløb [In Danish]. www.sst.dk: Sundhedsstyrelsen; 2020.

**Supplementary Table 2 - Codebook**

| Variable name | Category | Data type | Items | Response  categories  and labels |
| --- | --- | --- | --- | --- |
|  |  |  |  |  |
| Events in the diagnostic course | Patient hesitated | Reported by the GP (by survey answer) | The patient described that he/she had hesitated to see a GP | No (0), Yes (1) |
|  | No diagnostic investigation wanted | Reported by the GP (by survey answer) | The patient did not want a diagnostic investigation | No (0), Yes (1) |
|  | No follow-up | Reported by the GP (by survey answer) | The patient did not comply with the follow-up agreement | No (0), Yes (1) |
|  | Wait and see | Reported by the GP (by survey answer) | The GP advised to wait and see without a time indication | No (0), Yes (1) |
|  | Treatment or referral for another illness | Reported by the GP (by survey answer) | The GP treated or referred on suspicion of another illness than cancer first | No (0), Yes (1) |
|  | Normal tests | Reported by the GP (by survey answer) | The GP waited because of normal test results | No (0), Yes (1) |
|  | Suspicion of another cancer type | Reported by the GP (by survey answer) | The GP referred the patient on suspicion of another cancer type first | No (0), Yes (1) |
| The GP’s first referral | Cancer patient pathway | Reported by the GP (by survey answer) | Referred to specific cancer patient pathway first | No (0), Yes (1) |
|  | Non-specific Cancer Patient Pathway | Reported by the GP (by survey answer) | Referred to a Diagnostic Center (in a non-specific signs and symptoms of cancer-cancer patient pathway) first | No (0), Yes (1) |
|  | Diagnostic imaging | Reported by the GP (by survey answer) | Referred to diagnostic imaging first | No (0), Yes (1) |
|  | Specialist or another hospital department | Reported by the GP (by survey answer) | Referred to a specialist or another hospital department first | No (0), Yes (1) |
|  | Acute hospitalization | Reported by the GP (by survey answer) | Acutely hospitalized | No (0), Yes (1) |
| First place of contact | Out of hours service | Reported by the GP (by survey answer) | Where did the patient first turn to with symptoms or signs that you retrospectively believe could be due to the cancer: the out of hours service or another general practitioner than their regular | No (0), Yes (1) |
|  | Specialist | Reported by the GP (by survey answer) | Where did the patient first turn to with symptoms or signs that you retrospectively believe could be due to the cancer: a medical specialist (other than a general practitioner) | No (0), Yes (1) |
|  | Hospital | Reported by the GP (by survey answer) | Where did the patient first turn to with symptoms or signs that you retrospectively believe could be due to the cancer: the hospital, including emergency call/112, outpatient clinic and during hospitalization | No (0), Yes (1) |
|  | No symptoms | Reported by the GP (by survey answer) | No symptoms. The cancer was diagnosed based on screening (breast, colorectal or cervix) | No (0), Yes (1) |
|  | Unknown | Reported by the GP (by survey answer) | Unknown where the patient was first seen | No (0), Yes (1) |
|  | General practitioner | Reported by the GP (by survey answer) | Where did the patient first turn to with symptoms or signs that you retrospectively believe could be due to the cancer: their own general practitioner | No (0), Yes (1) |
| Explanatory variables: |  |  |  |  |
| Patient characteristics | Gender | Determined from the patient’s civil registration number | Female or male | Female (0), Male (1) |
|  | Age | Determined from the patient’s civil registration number | Age at the time of the cancer diagnosis | Continuous  For analyses categorized:  0-20, 21-40, 41-60, 61-80 and >80 |
| First symptoms that could be due to the cancer | Non-specific or general symptoms | Reported by the GP (by survey answer) | Non-specific or general symptoms as the presenting symptoms or signs that retrospectively could be due to the cancer | No (0), Yes (1) |
|  | Specific alarm symptoms | Reported by the GP (by survey answer) | Specific cancer symptoms as the presenting symptoms or signs that retrospectively could be due to the cancer | No (0), Yes (1) |
|  | None | Reported by the GP (by survey answer) | No symptoms as the presenting symptoms or signs that retrospectively could be due to the cancer (was diagnosed by a coincident finding in the examination, blood tests or diagnostic imaging) | No (0), Yes (1) |
|  | Do not know | Reported by the GP (by survey answer) | Unknown which symptoms were the presenting symptoms or signs that retrospectively could be due to the cancer | No (0), Yes (1) |
| Exclusion criteria |  | Reported by the GP (by survey answer) | The patient did not have a new cancer diagnosis at the time of diagnosis stated | No (0), Yes (1) |
|  |  | Reported by the GP (by survey answer) | Do not have any patient records | No (0), Yes (1) |

## **Supplementary Table 3. Descriptive characteristics of incident-CRC patients with first contact in general practice, and results of cluster regression analyses estimating the associations between age, sex, and symptom presentation and diagnostic process events, shown as Odds Ratios (ORs) with 95% confidence intervals (CIs)^1^, in 653 incident CRC patients in Danish general practice above 40 years (2019-2021)**

|  | **The patient hesitated with healthcare-seeking** | **The patient did not want an investi-gation** | **The patient did not comply with follow-up agreement** | **The GP advised watchful waiting with no time indication** | **The GP treated or referred on the suspicion of another disease first** | **The GP waited because of normal examina-tions** | **The GP referred to investi-gation on suspicion of another cancer type first** |
| --- | --- | --- | --- | --- | --- | --- | --- |
|  | OR (95% CI) | OR (95% CI) | OR (95% CI) | OR (95% CI) | OR (95% CI) | OR (95% CI) | OR (95% CI) |
| Sex |  |  |  |  |  |  |  |
| Women | Ref | Ref | Ref | Ref | Ref | Ref | Ref |
| Men | 0.90 (0.58,1.40) | 0.91 (0.53,1.55) | **2.87 (1.02,8.09)** | 1.35 (0.58,3.15) | 0.77 (0.53,1.11) | 0.84 (0.42,1.66) | 0.90 (0.44,1.82) |
| Age groups, years |  |  |  |  |  |  |  |
| 41-60 | Ref | Ref | Ref | Ref | Ref | Ref | Ref |
| 61-80 | 0.73 (0.38,1.39) | 1.16 (0.49,2.78) | 0.52 (0.17,1.62) | 0.97 (0.27,3.51) | 0.86 (0.48,1.52) | 1.30 (0.46,3.62) | 0.93 (0.29,2.97) |
| >80 | 0.87 (0.47,1.60) | **2.50 (1.05,5.96)** | 0.77 (0.25,2.41) | 2.02 (0.57,7.20) | 0.95 (0.51,1.76) | 1.19 (0.41,3.45) | 0.85 (0.23,3.18) |
| Symptom presentation |  |  |  |  |  |  |  |
| None | 0.48 (0.18,1.29) | 0.33 (0.07,1.53) | N/A | N/A | **0.24 (0.07,0.80)** | 0.40 (0.05,3.18) | N/A |
| Only specific | Ref | Ref | Ref | Ref | Ref | Ref | Ref |
| Only non-  specific | **0.49 (0.29,0.82)** | 0.74 (0.42,1.32) | 1.62 (0.63,4.15) | **2.53 (1.08,5.94)** | **2.53 (1.73,3.70)** | **2.10 (1.15,3.82)** | **5.87 (2.05,16.81)** |
| Both | 0.38 (0.13,1.10) | 0.71 (0.23,2.18) | 2.15 (0.54,8.53) | 2.43 (0.59,9.89) | 1.34 (0.67,2.65) | 0.83 (0.19,3.52) | **6.70 (1.68,26.72)** |

1 Adjusted for age, sex, and symptom presentation.

* The number of the different events do not sum up to 100% as it was also possible to indicate none of the abovementioned events.

**Supplementary Table 4 Results of cluster regression analyses estimating the associations between age, sex, and symptom presentation and the first referral in the diagnostic process, shown as Odds Ratio1 (Adj. OR) with 95% confidence intervals (CIs), in 653 incident CRC patients in Danish general practice above 40 years (2019-2021)**

|  | **Referred to a specific cancer patient pathway** | **Referred to a non-specific cancer patient pathway** | **Referred to diagnostic imaging** | **Referred to specialist or other hospital department** | **Acute hospitalization** |
| --- | --- | --- | --- | --- | --- |
|  | OR (95% CI) | OR (95% CI) | OR (95% CI) | OR (95% CI) | OR (95% CI) |
| Sex |  |  |  |  |  |
| Women | Ref | Ref | Ref | Ref | Ref |
| Men | 1.26 (0.94,1.68) | 0.82 (0.43,1.55) | 1.36 (0.82,2.26) | **0.68 (0.47,0.99)** | 0.95 (0.60,1.50) |
| Age groups, years |  |  |  |  |  |
| 41-60 | Ref | Ref | Ref | Ref | Ref |
| 61-80 | 1.27 (0.74,2.17) | 2.11 (0.61,7.34) | 1.26 (0.56,2.84) | 0.74 (0.39,1.40) | 0.65 (0.35,1.22) |
| >80 | 0.98 (0.57,1.70) | 1.49 (0.38,5.81) | 1.46 (0.57,3.77) | 0.52 (0.25,1.10) | 1.43 (0.74,2.74) |
| Symptom presentation |  |  |  |  |  |
| None | **0.38 (0.22,0.66)** | **3.66 (1.20,11.13)** | 0.91 (0.25,3.33) | 1.12 (0.53,2.39) | 1.82 (0.83,3.98) |
| Only specific | Ref | Ref | Ref | Ref | Ref |
| Only non-  Specific | **0.27 (0.19,0.39)** | 2.40 (0.95,6.09) | **3.10 (1.63,5.87)** | 1.11 (0.73,1.69) | **2.63 (1.55,4.49)** |
| Both | 0.75 (0.43,1.33) | 3.74 (1.20,11.67) | 0.93 (0.28,3.11) | 0.85 (0.44,1.66) | 1.59 (0.66,3.83) |

^1^ Adjusted for age, sex, and symptom presentation.

* The number of the different processes do not sum up to 100% as it was also possible to indicate none of the abovementioned processes
